# Cerebrovascular manifestations in hematological diseases: an update

**DOI:** 10.1007/s00415-021-10441-9

**Published:** 2021-02-13

**Authors:** José M. Ferro, Joana Infante

**Affiliations:** 1 Serviço de Neurologia, Departamento de Neurociências e Saúde Mental, Hospital de Santa Maria, Centro Hospitalar Lisboa Norte, Lisbon, Portugal; 2 Faculdade de Medicina, Universidade de Lisboa, Hospital de Santa Maria, Neurology, 6th Floor, Avenida Professor Egas Moniz s/n, 1649-035 Lisbon, Portugal; 3 Serviço de Hematologia e Transplantação de Medula, Hospital de Santa Maria, Centro Hospitalar Lisboa Norte, Lisbon, Portugal

**Keywords:** Stroke, Intracerebral hemorrhage, Cerebral venous thrombosis, PRES, Myeloproliferative neoplasm, Polycythaemia vera, Essential thrombocythemia, Leukemia, Lymphoma, Myeloma, POEMS, Sickle cell disease, Paroxysmal nocturnal hemoglobinuria, Thrombotic thrombocytopenic purpura

## Abstract

Patients with hematological diseases often experience cerebrovascular complications including ischemic stroke, intracerebral and subarachnoid hemorrhage, microbleeds, posterior reversible encephalopathy syndrome, and dural sinus and cerebral vein thrombosis (CVT). In this update, we will review recent advances in the management of cerebrovascular diseases in the context of myeloproliferative neoplasms, leukemias, lymphomas, multiple myeloma, POEMS, paroxysmal nocturnal hemoglobinuria (PNH), thrombotic thrombocytopenic purpura (TTP), and sickle-cell disease. In acute ischemic stroke associated with hematological diseases, thrombectomy can in general be applied if there is a large vessel occlusion. Intravenous thrombolysis can be used in myeloproliferative neoplasms and sickle-cell anemia, but in other diseases, a case-by-case evaluation of the bleeding risks is mandatory. Patients with sickle-cell disease and acute stroke need very often to be transfused. In PNH, acute ischemic stroke patients must be anticoagulated. Most patients with CVT can be treated with low-molecular weight heparin (LMWH) acutely, even those with leukemias. Prevention of recurrence of cerebral thrombotic events depends on the control of the underlying disease, combined in some conditions with antithrombotic drugs. The recent introduction of specific monoclonal antibodies in the treatment of PHN and TTP has dramatically reduced the risk of arterial and venous thrombosis.

## Introduction

In registries of busy comprehensive stroke centers, hematological diseases are a very rare cause of stroke, in particular of ischemic stroke [1, 2]. However, patients with hematological diseases often experience cerebrovascular complications including ischemic stroke, intracerebral and subarachnoid hemorrhage, microbleeds, posterior reversible encephalopathy syndrome (PRES), and dural sinus and cerebral vein thrombosis (CVT) [3]. In this review, we will update the readers on recent advances in the epidemiology, pathogenesis, better recognized clinical features, investigation, acute management, and secondary prevention of cerebrovascular diseases in the context of some hematological illnesses. We selected for this review myeloproliferative neoplasms, leukemias, lymphomas, multiple myeloma, paroxysmal nocturnal hemoglobinuria, thrombotic thrombocytopenic purpura, and sickle-cell disease.

## Myeloproliferative neoplasms

The myeloproliferative neoplasms (MPN) are a group of diseases in which there is an increased proliferation of one or more subtypes of myeloid cells [4]. Those associated with stroke are polycythemia vera (PV) and essential thrombocythemia (ET) [5]. The Janus kinase *JAK2* exon 14 V617F mutation is present in more than 95% of patients with PV and in approximately 50% of those with ET. Other mutually exclusive driver mutations are found in the genes *MPL*, *CALR*, and *JAK2* exon 12. The incidence of MPN increases exponentially after the age of 60. MPN are chronic diseases with years of survival, estimated at 14 years for PV and 20 years for ET [4, 6].

Thrombotic complications are frequent in MPN. A recent large study found that the 5-year risk of vascular disease ranged from 0.5 to 7.7% in patients with MPNs, with adjusted HRs which were 1.3–3.7-fold higher than in the general population [7] Stroke can be the first clinical manifestation of an underlying MPN. In the National Inpatient Sample, a total of 552,422 hospitalizations involved patients with a diagnosis of ET, 20,650 of which were due to stroke (0.04%) [8]. Clinical hints suggesting MPN as the cause of stroke are multiple or recurrent territorial infarcts [9], often with large vessel occlusion. Transient ischemic attacks (TIAs) in the context of MPN are usually multiple and may be typical or atypical (isolated dysarthria, diplopia or unsteadiness, hearing loss, transient focal deficits with prominent headache, and very brief or sequential deficits). Erythromelalgia (a burning or painful sensation of the fingers, palms, and sometimes also of the toes and soles accompanied by a red-cyanotic skin discoloration of the affected areas) and splenomegaly are suggestive systemic findings. World Health Organization 2016 diagnostic laboratory criteria [10] for PV require sustained (> 1 month) increased hemoglobin or hematocrit values (hemoglobin > 16.5 g/dL in men, > 16.0 g/dL in women; hematocrit > 49% in men, > 48% in women) with subnormal erythropoietin levels and the absence of a secondary cause of erythrocytosis. ET sustained high platelet counts (> 450,000/µL) (> 1 month) in the absence of a secondary cause of thrombocytosis. The *JAK2* V617F mutation is present in the majority of stroke patients. In half of the patients, there is an additional stroke risk factor, such as atherosclerosis, dissection, or atrial fibrillation [11]. Female gender, atrial fibrillation, stroke, higher comorbidity score, and age 80 or more are independent predictors of mortality in stroke associated with ET [8].

**Fig. 1:**
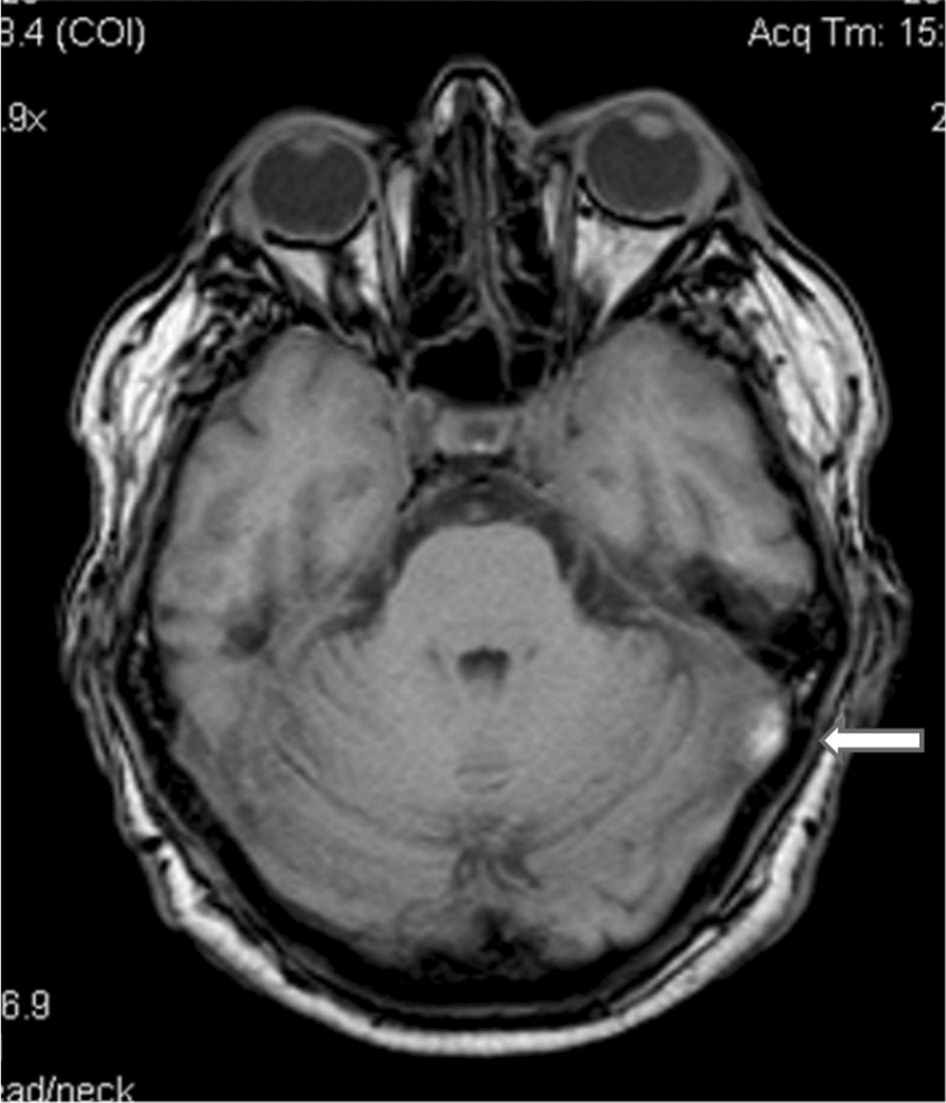
Thrombosis of the left lateral sinus (arrow) with a 67 year old male with essential thrombocythemia, presenting with headache and papilledema

MPN should also be considered as a possible cause of cerebral venous thrombosis in middle-aged/elderly patients (Fig. 1). MPN are also a risk factor for CVT recurrence [12]. Hemorrhagic stroke is very rare in MPN, but may be fatal. A recent report confirmed the occurrence of intracerebral hemorrhages and subarachnoid convexity hemorrhage in MPN [13, 14].

The current algorithm treatment for patients with PV and ET recommends a careful assessment of cardiovascular risk factors and the use of low-dose aspirin (80–100 mg), unless contraindicated. Erythromelalgia and atypical TIAs also respond to aspirin, which is an irreversible inhibitor of platelet COX-1 activity. Antiplatelet drugs with other mechanisms of action are ineffective. Twice-daily aspirin dosing may be considered in low-risk patients whose microvascular symptoms are not adequately controlled with once-daily dosing or in those with cardiovascular risk factors and a leukocytosis (> 15,000/μL) [15, 16]. In PV, erythrocytosis should be controlled by phlebotomy to maintain a hematocrit below 45%.

The risk of thrombotic events modulates the need for cytoreductive therapy, in addition to aspirin and phlebotomies. In high-risk PV patients for thrombotic events, defined as having a prior thrombotic event (e.g., stroke) and/or age > 60 years, pharmacological therapy is needed to lower and control the red blood cell. Other risk factors in high-risk PV patients include other vascular risk factors for ischemic stroke or CVT, and *JAK2* mutations. Hydroxyurea is the most commonly used cytoreductive drug. Busulfan is an alternative in older patients, while younger patients may receive interferon *α* and/or anagrelide. Recently, effective and nongenotoxic JAK inhibitors (ruxolitinib and fedratinib) have been developed and may be used in patients refractory to first-line therapies.

Similarly, the mainstay of ET management includes cardiovascular risk assessment and aspirin as described for PV. Cytoreduction is reserved to lower the platelet count in some patients with intermediate and all with high-risk disease, including those with previous stroke [16]. With very high platelet counts (> 1,000,000/µL), an acquired von Willebrand syndrome can develop and bleeding may occur. In this acquired deficiency of von Willebrand factor, aspirin is contraindicated until platelet count is reduced to < 1,000,000/µL by cytoreduction.

In patients with PV or ET and an acute stroke, the recommendations for the treatment of the different types of stroke should be followed. Platelet counts should be monitored frequently during unfractionated heparin treatment, to detect heparin-induced thrombocytopenia. For patients with cerebral venous thrombosis, oral anticoagulation should be given for at least 6 months, unless contraindicated [17]. There is not yet high-quality evidence to support the use of DOACs in MPN, but randomized-controlled trials are ongoing.

## Leukemias

**Fig. 2:**
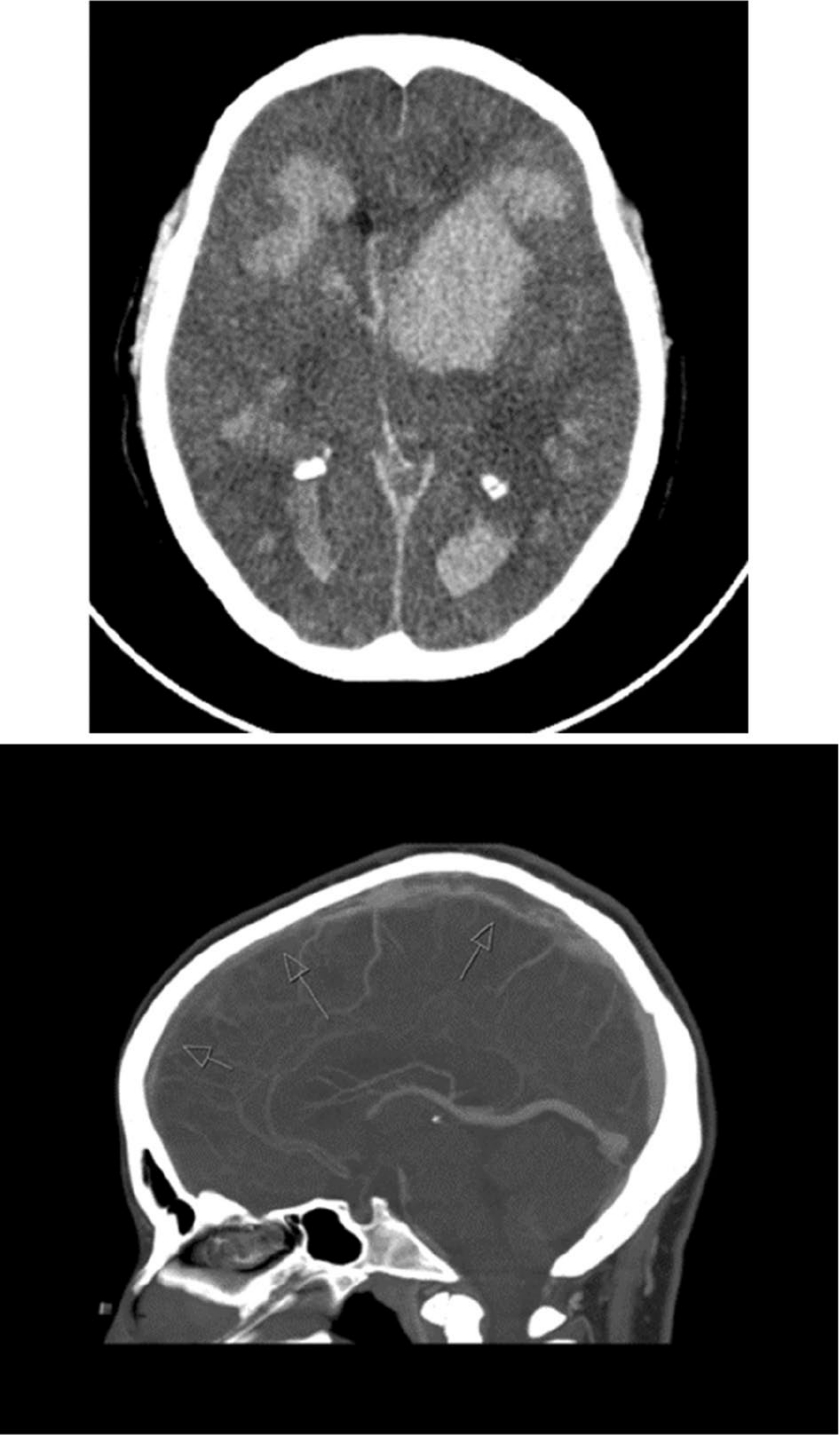
Stroke in acute leukemias: multiple hemorrhages in acute myeloid leukemia with hyperleukocytosis (up); cerebral venous thrombosis in acute monocytic leukemia (bottom)

Leukemias, especially acute leukemias, can cause stroke, both arterial and venous, ischemic or hemorrhagic [18] (Fig. 2). Acute leukemias are a relevant cause of stroke in children and young adults, and while it is usually a complication after diagnosis, stroke can occasionally be a presenting feature. In such rare instances, the key clinical hint in the hyperacute phase of stroke is the blood cell count, showing anemia and thrombocytopenia and circulating immature leukocytes, which can be observed on peripheral blood smear, and frequently result in increased white blood cell counts. Mechanisms of ischemic stroke in leukemias include hypercoagulability, disseminated intravascular coagulopathy (DIC), leukostasis in leukemias with hyperleukocytosis (> 50 × 109/L) (Fig. 1) marantic endocarditis, paradoxical embolism, atrial fibrillation and other traditional vascular risk factors, infections, and also cancer therapy (radiation, chemotherapy, immunotherapy, and transplantation). Leukostasis is an emergency, needing hydration and chemotherapy, or leukapheresis as additional option.

Intracranial bleeding is a serious, potentially fatal, cerebrovascular complication in patients with acute leukemia. Bleeding can be epi or subdural, subarachnoid, intraventricular, and intracerebral, and often occurs at multiple locations. Thrombocytopenia is the main risk factor for intracranial bleeding. A recent systematic review of intracerebral hemorrhages in patients with hematological malignancies, reported that the median survival for patients with intracerebral hemorrhages ranged from 20 days to 1.5 months. The median survival for the subset of patients having intracerebral hemorrhage within 10 days of diagnosis of hematologic malignancy was only 5 days. Intraparenchymal hemorrhages, multiple foci of hemorrhage, transfusion-refractory low platelet counts, leukocytosis, low Glasgow Coma Scale scores at presentation, and intracerebral hemorrhage early in the treatment course were all associated with worse outcomes [19]. Intracerebral hemorrhage is particularly common in acute promyelocytic leukemia (APL). APL is characterized by the PML–RARa fusion in leukemia cells, caused by *t*(15;17), and a thrombo-hemorrhagic syndrome including DIC and secondary hyperfibrinolysis which frequently results in bleeding, but can also cause thrombosis [20]. The early institution of all-trans retinoic acid and intensive support with fresh-frozen plasma, fibrinogen, and/or platelets have improved outcomes and the early hemorrhagic death rate, which nevertheless is still significant.

Leukemias, especially acute lymphoblastic leukemia (ALL) and APL, can also be complicated by cerebral venous thrombosis (CVT). In fact, the most common cause of cerebral infarction at, or shortly after, the diagnosis of ALL is CVT. CVT occurs both in children [21, 22] and adults [19]. CVT is usually due to leukemic infiltration of the superior sagittal sinus. Risk factors for CVT in patients with ALL include treatment with l-asparaginase, high-dose steroid regimens, and intrathecal methotrexate [21–25]. A few cases have recently been described following placement of peripherally inserted central catheters for starting chemotherapy in ALL [26]. Treatment with all-trans retinoic acid is the major risk factor for CVT in acute promyelocytic leukemia [27]. The majority of reported children and young adults with leukemia and CVT were treated acutely with LMWH, without bleeding complications [21, 25]. In a recent single-center CVT case series, from the 111 CVT adult cases diagnosed, only seven coexisted with hematological malignancy (lymphoma, leukemia, multiple myeloma, and myelodysplastic syndrome). Other prothrombotic conditions were present in all cases. Several anticoagulant strategies were used during the acute phase, after which five patients remained on warfarin indefinitely. One patient died due to cerebral hemorrhage in the acute phase, as a complication of their anticoagulation. In the remaining six patients, there was no recurrence of CVT or other complications from anticoagulation, suggesting that most patients benefit from the long-term anticoagulation option. A multidisciplinary approach is paramount in making decisions regarding the time and type of anticoagulation [28]. Endovascular mechanical thrombectomy is an alternative to anticoagulation in severe cases of CVT or if anticoagulation is prohibited due to severe thrombocytopenia or bleeding dyscrasia [29]. In patients with severe thrombocytopenia (< 50 × 10^9^/L), therapeutic doses of anticoagulation may be considered, with platelet transfusion support to maintain platelet counts above 40–50 × 10^9^/L [30].

The International Society on Thrombosis and Hemostasis issued in 2020 guidance on the prevention and management of asparaginase-related venous thromboembolism [31]. Asparaginase contributes to a hypercoagulable state through decreased production of natural anticoagulants (antithrombin, protein C and S). For CVT, the International Society on Thrombosis and Hemostasis suggests LMWH in the acute phase, followed by DOACs, after resolution of severe thrombocytopenia (< 50 × 10^9^/L). Short-term administration of antithrombin concentrate is also suggested. If patients are not considered to be at increased risk of hemorrhage, it is suggested to continue anticoagulation until completion of the chemotherapy or achievement of complete remission. Resuming asparaginase treatment may be considered after stabilization of CVT, but the evidence considering the safety of this option is very limited.

## Lymphomas

The mechanisms of ischemic and hemorrhagic stroke are rather similar in leukemias and lymphomas. Additional ischemic stroke mechanisms in lymphomas are vasculitis, thrombotic microangiopathy, compression of vessels by lymphadenopathy, and intravascular lymphoma. In older patients with non-Hodgkin lymphoma, atrial fibrillation and other cardioembolic sources along with cervical or intracranial atheroma should be excluded before attributing the stroke to the lymphoma. Among patients treated for cancer and diagnosed at < 40 years of age, the majority of strokes during long-term follow-up occur in patients treated for lymphomas [32].

## Multiple myeloma

Patients with multiple myeloma (MM) are at increased risk of ischemic and hemorrhagic stroke and cerebral venous thrombosis [28, 33]. In MM, ischemic stroke can occur through several pathophysiological mechanisms including hypercoagulability (e.g., abnormal plasma thrombin generation, microparticle-associated tissue factor, high PAI-1 levels, increased P-selectin, and acquired activated Protein C resistance) [34, 35] hyperviscosity, thrombotic microangiopathy, embolism from an amyloid cardiomyopathy and thrombogenic side-effects of medications such as lenalidomide [36], thalidomide, bortezomib, carfilzomib [37], dexamethasone, and erythropoiesis-stimulating agents [38].

Two recent studies provided more evidence on the risk and predictors of stroke in MM patients. In a study including 395 consecutive patients (median age of 70 years) with newly diagnosed symptomatic MM, with a median follow-up period of 18 months, 16 patients suffered a stroke (10 ischemic strokes and 6 hemorrhagic) (5-year cumulative incidence rate, 7.45%). The *κ* light chain isotype, previous stroke, and serum creatinine > 2 mg/dL were independent risk factors considering all strokes. While atrial fibrillation and previous stroke were significant risk factors for ischemic stroke, and serum creatinine > 2 mg/dL and previous stroke were significant risk factors for hemorrhagic stroke [39]. Of 1148 MM patients enrolled in Total Therapy protocols, 46 developed a cerebrovascular event (ischemic stroke, 33; transient ischemic attack, 11; intracerebral hemorrhage, 2). Renal insufficiency and multiple myeloma stages I and II were independent predictors of stroke. Hypercoagulability, atrial fibrillation, and small-vessel occlusion were the most common mechanisms of stroke [40]. This study also confirmed that in elderly patients with multiple myeloma, stroke may be due to “usual” stroke mechanisms. Therefore, cardioembolism from atrial fibrillation or other major cardiac embolic sources, and significant carotid or intracranial atheroma, should always be ruled out before attributing stroke to MM or its treatments.

Immunomodulatory drugs (lenalidomide, thalidomide, pomalidomide) are widely used in MM, and they confer a higher risk of venous thromboembolism. Therefore, some form of thromboprophylaxis is mandatory, while patients are on immunomodulatory therapy, either with aspirin, for patients with low risk of venous thromboembolism, or LMWH for those with higher risk [41]. Apixaban has also been used with encouraging results.

## Polyneuropathy, organomegaly, endocrinopathy, monoclonal protein, and skin changes syndrome (POEMS)

POEMS syndrome (an acronym for polyneuropathy, organomegaly, endocrinopathy, monoclonal protein, and skin changes) is a paraneoplastic phenomenon resulting from an underlying plasma cell neoplasm. Not all five features must be present to make the diagnosis, although peripheral neuropathy and a monoclonal plasma cell disorder are mandatory. There is a range of other features not captured in the acronym, including myeloproliferation manifested by polycythemia/thrombocytosis during the phase of active disease. Disease activity largely correlates with vascular endothelial growth factor (VEGF) levels in the blood [42].

Ischemic stroke is a well-known complication in patients with POEMS. Three recent case series confirmed the typical features of stroke associated with POEMS [43–45]. The frequency of ischemic stroke among patients with POEMS was 8%. Infarcts were usually multiple. Most patients had extra or intracranial arterial segmental stenosis, often in multiple vessels. Almost all strokes occur before or within 3 months of the diagnosis of POEMS, mostly before the start of chemotherapy. Patients with ischemic stroke were older, had a higher level of fibrinogen and a lower survival rate than those without stroke [44]. As in MM, strokes induced by lenalidomide have been also described [46]. The increased prevalence of thromboembolic events in POEMS syndrome (30%) mandates a careful thrombotic risk assessment and thromboprophylaxis, especially if patients are under treatment with an immunomodulatory drug. An empirical thromboprophylaxis regimen with LMWH plus antiplatelet agent is advocated, from diagnosis until serum VEGF < 1000 µg/mL, when the antiplatelet drug can be stopped. LMWH is continued until the disease is in remission [45].

The vasculopathy of POEMS affects multiple large extra and/or intracranial vessels. A few reported patients had angiographic features suggesting inflammation, such as vessel irregularities and beading in the distal intracranial arteries [47], or enhancement of the thickened wall of the internal carotid on gadolinium-enhanced images [44, 48]. Cases with a Moyamoya pattern were recently reported [49, 50]. One of these cases had a post-mortem study, which revealed duplication of the internal elastic lamina, intima deposits of mucopolysaccharides, and strongly increased *α*-smooth-muscle actin (a marker of smooth-muscle cells), but no evidence of inflammation or vasculitis [50]. POEMS cerebral vasculopathy appears to be a progressive obliterative arteriopathy driven by the continuous stimulation through overproduction of VEGF.

## Paroxysmal nocturnal hemoglobinuria

Paroxysmal nocturnal hemoglobinuria (PNH) is a rare clonal haematopoietic stem cell disease characterized by a triad of episodes of intravascular hemolytic anemia, thrombosis, and aplastic anemia, with a risk of evolution to myelodysplastic syndromes or acute myeloid leukemia. PNH is caused by somatic mutations in phosphatidylinositol glycan (GPI) anchor biosynthesis class A gene (*PIGA*), causing deficiency of GPI-anchored proteins, including CD55 and CD59, which are complement inhibitors. Clinical manifestations occur when a haematopoietic stem cell clone with *PIGA* mutations generates mature blood cells that are deficient in GPI-anchored proteins. Loss of CD55 and CD59 renders PNH erythrocytes susceptible to complement-mediated intravascular hemolysis, which can lead to thrombosis through the release of free hemoglobin and procoagulant microparticles. Thrombosis usually occurs in atypical locations (e.g., abdominal, hepatic, and cerebral), venous thrombosis being more common than arterial. Venous thrombosis is the leading cause of mortality in PNH. PNH has a highly variable natural history and is associated with significant morbidity. In suspected cases, complete cell blood counts and smear, reticulocytes and LDH, and urinalysis should be requested. The diagnosis is confirmed by peripheral blood flow cytometry, which will show an absence or severely decreased GPI-anchored proteins on > 1 lineages of blood cells [51]. A recent study suggests that whole-body MRI may be useful to assess the complete vascular status of PNH patients and allow for the detection of previously undiagnosed vascular complications [52].

**Fig. 3:**
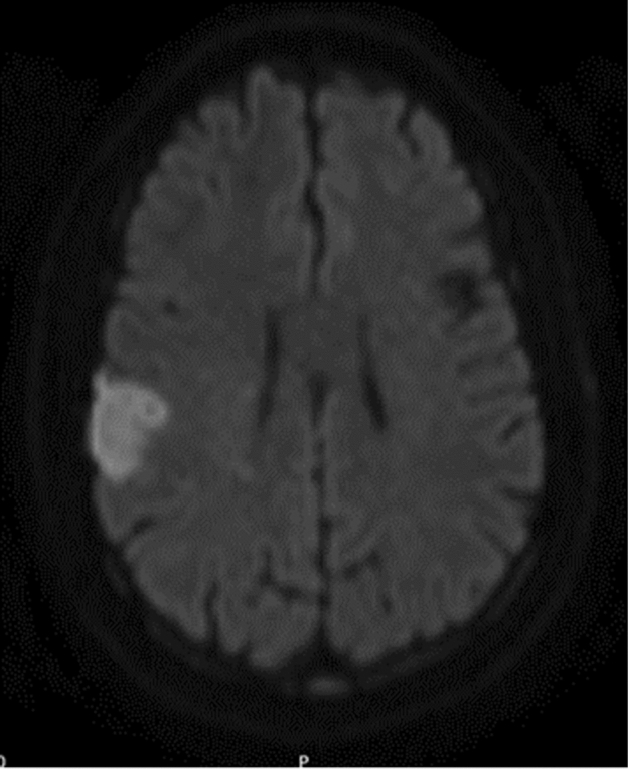
Acute stroke in a patient with paroxysmal nocturnal hemoglobinuria, showing an right hemispheric acute and a left frontal chronic infarct on MR DWI sequence

The spectrum of cerebrovascular disease in PNH comprises cerebral venous thrombosis (CVT), single or multiple TIA and cortical [53] or lacunar [54] ischemic stokes (Fig. 3), and other rarer manifestation such as Moyamoya syndrome [55] and PRES [56]. In asymptomatic PNH patients, brain MRI may disclose pathological findings, including white matter lesions, infarcts, and microbleeds. Compared with age- and sex-matched controls, PNH patients show an increased frequency of periventricular white matter lesions (32 vs 5.2%) and of severe white matter lesions (26 vs 2.6%). Prior partial cerebral venous thrombosis have been also observed in PNH cases [57].

Cerebral venous thrombosis is the second most frequent location of venous thrombosis after hepatic veins and occurs in 2–8% of PHN patients. Nevertheless, it is a very rare cause of CVT (< 1%). The largest case series of CVT in PNH is a multicentre French study of 15 patients, observed from 1990 to 2012. Females predominate (12/15) and half of them presented with associated hormonal risk factors. Three patients had concomitant hepatic vein thrombosis, while in four cases, CVT was the first manifestation of PNH. The clinical hints to the diagnosis of PNH were cytopenia, hemolysis, and abdominal thrombosis. There was no major difference in CVT characteristics compared with non-PNH CVT cases, except for a younger age at diagnosis in PNH patients. All patients were treated with anticoagulation therapy. One patient died in the acute phase, but all surviving patients were living independently at 1 year. The recurrent thrombosis rate at 6 years was 50%, occurring in patients who did not have bone marrow transplantation or eculizumab therapy. Median survival time was 9 years. Cases of death were mainly related to hepatic vein thrombosis [58].

Therapeutic strategies for PNH are bone marrow transplantation as the only curative treatment for the bone marrow failure component of disease, and monoclonal antibody complement inhibitors, such as eculizumab (anti-C5 monoclonal antibody), which controls complement-mediated intravascular hemolysis, reduces the risk of recurrent arterial and venous thrombosis, and prolongs survival [51]. Eculizumab opened a new era in the treatment of NPH. Unfortunately, access to eculizumab is often hampered by its very high cost. Patients who cannot access eculizumab or those with a suboptimal response to eculizumab may also be considered for transplantation.

Venous or arterial thrombosis requires immediate full anticoagulation with UFH/LMWH, followed by prolonged oral anticoagulation with vitamin K antagonists. In the acute thrombosis setting, eculizumab should be started in the first 24 h to reduce extension of the thrombus and thrombosis recurrence [51]. In case of severe thrombocytopenia, platelet transfusion is needed to increase the platelet count to a target of > 30–50,000/µL, before anticoagulation is started.

## Thrombotic thrombocytopenic purpura

Thrombotic thrombocytopenic purpura (TTP) is due to a genetic or acquired severe deficiency of the von Willebrand factor-cleaving serine protease ADAMTS13 (a disintegrin and metalloproteinase with a thrombospondin type 1 motif, member 13), allowing unrestrained adhesion of the von Willebrand factor multimers to platelets and microthrombi formation, resulting in thrombocytopenia, hemolytic anemia, and tissue ischemia, mostly in the kidney and brain. The most frequent form of TTP is acquired immune-mediated (iTTP). TTP is a severe, relapsing life-threatening thrombotic microangiopathy, which critically depends on the clinician making a rapid diagnosis and starting treatment as soon as possible. The diagnosis is suspected on clinical (classical pentad of fever, microangiopathic hemolytic anemia, thrombocytopenia, renal dysfunction, and changes in mental status) and laboratory (anemia with schistocytes, increased reticulocytes (> 2.5%), thrombocytopenia, indirect (unconjugated) bilirubin > 2 mg/dL, undetectable haptoglobin, high lactate dehydrogenase) grounds and confirmed by very low levels (< 10%) of ADAMTS3 [59]. TTP causes a thrombotic microangiopathy with diffuse microthrombi formation in the microcirculation and hypoperfusion. Neurological involvement manifests as a stroke/TIA or as an encephalopathy with vigilance and mental status disturbances and/or seizures. Survival in iTTP has improved significantly since the introduction of plasma exchange as standard therapy, combined with immunosuppression with steroids, rituximab, cyclophosphamide, or vincristine. Recently, the addition of caplacizumab, an anti-von Willebrand factor humanized immunoglobulin fragment, to plasma exchange and immunosuppression, leads to reduced incidence of TTP-related death, TTP recurrence, stroke, and other thromboembolic events [60]. We do not use aspirin, as there is limited evidence on its efficacy and safety. However, aspirin, is suggested by some experts, if platelet counts are above 50,000/µL [61]. Platelet transfusions may cause a slightly increased risk of thrombosis in patients with TTP. Prophylactic platelet transfusions are not used in patients with TTP in the absence of severe bleeding or a required invasive procedure.

TTP is a rare cause of stroke among young adults [62]. In a literature review, only 17 cases were found (14 females; mean age 41 years). None of the patients had the classical pentad of TTP. Only 41% had a combination of thrombocythemia and hemolysis. Stroke was multifocal in 35% and included large artery strokes. No adverse event was observed following intravenous thrombolysis. Refractory and relapsing forms were observed in 47% [62].

Stroke can also occur after recovery from acute iTTP and is associated with reduced ADAMTS13 activity during remission. Low-ADAMTS13 is a vascular risk factors for stroke recurrence. Eighteen of 137 patients (13.1%) developed a stroke unrelated to an acute TTP episode over 3.08 years of follow-up (5 times higher than the expected prevalence). Stroke after recovery from acute TTP occurred in 0% (0 of 22) of patients with normal remission ADAMTS13 activity (> 70%) and in 27.6% (8 of 29) of patients with low ADAMTS13 activity (≤ 70%) [63].

Inherited or congenital TTP is extremely rare, but can be a cause of stroke [64]. In an international registry of congenital PTT, 25 patients (21%) suffered a stroke and 12 (10%) a TIA [65]. In a UK registry of 73 cases, 8% had TIAs and 19% strokes (ref). Regular infusions of fresh-frozen plasma significantly reduced the risk of stroke recurrence [66].

**Fig. 4:**
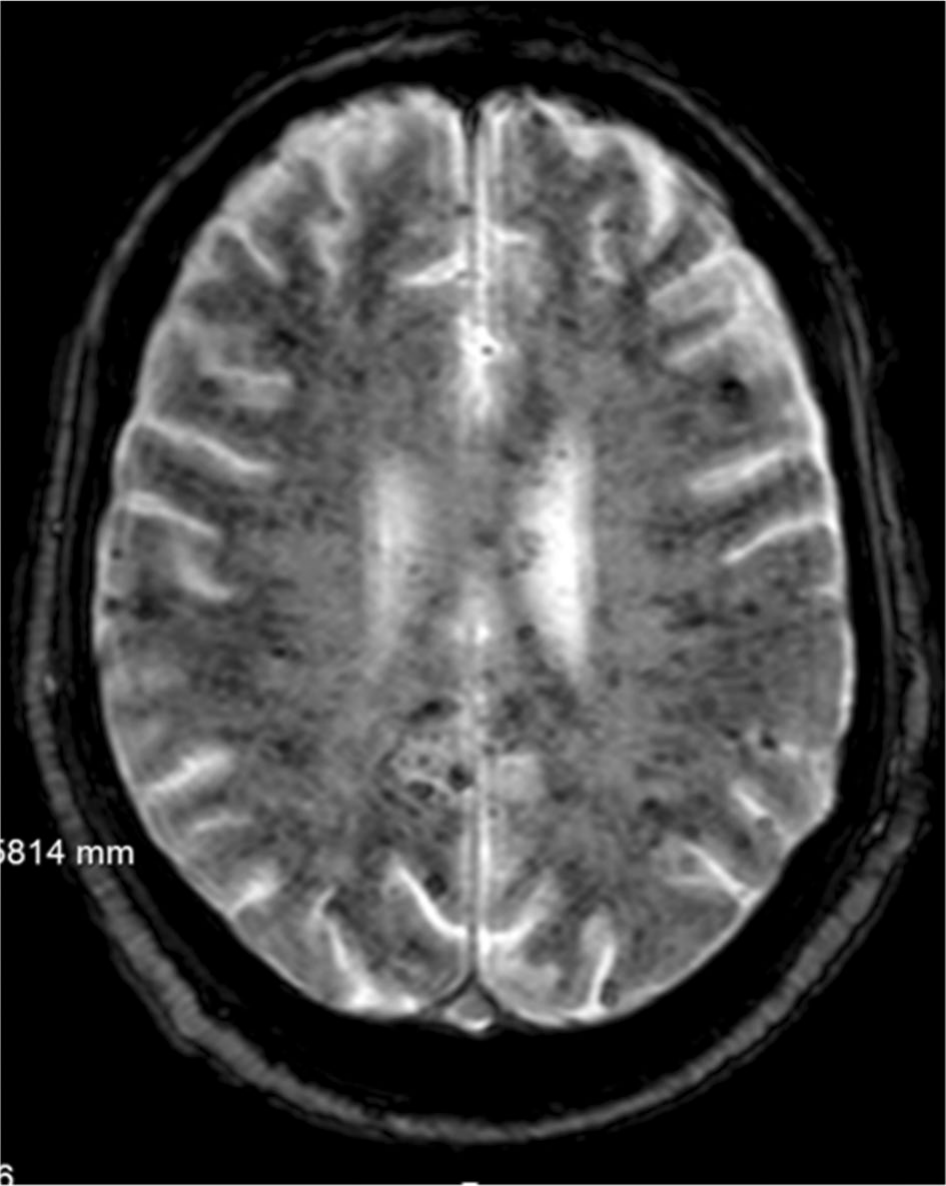
Uncountable microbleeds in a fatal case of thrombotic thrombocytopenic purpura

A case of PRES in a diabetic patient with acquired TTP has also recently been reported. The patient was normotensive, but had elevated blood urea and recent surgery for digital gangrene [67]. Patients with TTP can also develop multiple multifocal cerebral microbleeds (Fig. 4) on brain magnetic resonance imaging [68, 69].

## Sickle cell anemia

Cerebrovascular disease is very common in sickle-cell anemia (SCA) and a major cause of morbidity and cognitive impairment in both children and adults [70–72]. The main mechanism leading to cerebral infarction is abnormal erythrocyte adherence to the vascular endothelium and hemolysis, causing platelet aggregation and increased vasomotor tone, finally leading to thrombosis. In later stages of the disease, luminal narrowing occurs secondary to proliferation of smooth-muscle cells and fibroblasts within the intimal layer, the end result being an occlusive vasculopathy. Decreased arterial oxygen content also plays a role [73]. A Moyamoya angiopathy may develop in adults, which may lead both to ischemic and hemorrhagic stroke. Less frequent mechanisms of stroke are cardioembolism, fat embolism, dissection, and cerebral venous thrombosis [74]. Recently, emphasis has been put on silent cerebral infarctions, which occur in half of adults by age 30. Silent cerebral infarctions involve predominantly the white matter of the internal border zone [75]. Silent infarcts predict an increased risk for stroke, cognitive impairment, and failure to meet academic milestones.

The American Society of Hematology issued in 2020 new guidelines on the prevention, diagnosis, and treatment of cerebrovascular disease in children and adults with sickle-cell anemia [76]. An annual transcranial Doppler (TCD) and 1-time MRI screening are recommended in children with HbSS aged 2–16 years, to detect increased intracranial arterial flow velocities and fixed arteriopathy and/or silent cerebral infarcts, respectively. Children with abnormal TCD velocities are recommended to undergo regular blood transfusions for at least a year, with the goal of maintaining maximum HbS levels below 30% and hemoglobin levels above 9.0 g/dL to reduce the risk of stroke. In regions where regular blood transfusion are not available, hydroxyurea at a dosage at least 20 mg/kg daily should be initiated [77]. After 1 year, hydroxyurea can replace regular blood transfusion therapy in children with abnormal TCD results, provided that there is no MRA-defined vasculopathy or silent cerebral infarcts. In such cases, blood transfusions should be maintained indefinitely [76].

Concerning the treatment of an acute cerebrovascular event (stroke or TIA) in an adult with HbSS, emergent CT and CTA should be performed, oxygen supplementation is recommended, and exchange blood transfusion should be started immediately upon recognition of symptoms (ideally within 2 h of acute neurological symptom onset) if the Hb is under 8.5 g/dL aiming for a target Hb > 10 g/dL. Exchange transfusion is preferable vs simple transfusion, aiming to reach HbS level < 15–20%. If Hb is > 8.5 g/dL, exchange transfusion is suggested to decrease the possibility of a hyperviscosity syndrome. Intravenous alteplase (rtPA) in adults with HbSS presenting within < 4.5 h of onset of stroke symptoms can be used, as for patients without SCA. In fact, from 2,016,652 stroke patients admitted to Get With The Guidelines-Stroke sites in the US, a study comparing 832 SCA and 3328 non-SCA controls found no differences in admission variables, on the % use of thrombolytic therapy (8.2% for SCA vs 9.4% non-SCA) or in a prespecified set of outcome measures. These results indicate that coexistent SCA had no significant impact on the safety or outcome of thrombolytic therapy in acute ischemic stroke [78]. Nevertheless, IV rtPA should not delay exchange blood transfusion. If ischemic stroke is judged to be provoked by a cause other than SCA (e.g., cardiac or arterial emboli), rtPA can be provided first [76]. If, in an adult HbSS acute stroke patient, CTA identifies a large vessel occlusion and excludes Moyamoya, endovascular thrombectomy may be performed as for non-HbSS patients, although the accumulated experience of thrombectomy in HbSS patients is still very limited.

For secondary stroke prevention, adults with HbSS should receive stroke-modifiable risk factor control, antiplatelets, and statins such as that recommended for non-HbSS stroke patients, together with regular simple blood transfusions. Hydroxyurea has been demonstrated to be inferior to transfusions for this indication [79]. Patients refractory to these treatments, may be considered for hematopoietic stem cell transplantation. Patients with Moyamoya arteriopathy may benefit from revascularization neurosurgery [80]. Patients with silent cerebral infarcts should be managed similarly to those who had suffered a clinically apparent stroke, namely with regular blood transfusions. Periodic (12–24 months) neurological and cognitive assessments and brain MRI are also recommended [76].

## Cerebrovascular complications of treatments of hematological diseases

### Posterior reversible encephalopathy syndrome

Almost all drugs used in the induction or maintenance treatment phases of leukemias and lymphomas have been reported to be associated with PRES. In an MEDLINE search of PRES and 65 drugs used to treat hematological disease and PRES, we retrieved reports of PRES associated with the use (single or combined) of IT-methotrexate, cytarabine, cyclophosphamide, vincristine, vinblastine, idarubicin, dacarbazine, bleomycin, asparaginase, cisplatin, carboplatin, fludarabine, nivolumab, brentuximab, pembrolizumab, eculizumab, gilteritinib, etoposide, thalidomide, bortezomib, carfilzomib, rituximab, cyclosporine, and tacrolimus.

**Fig. 5:**
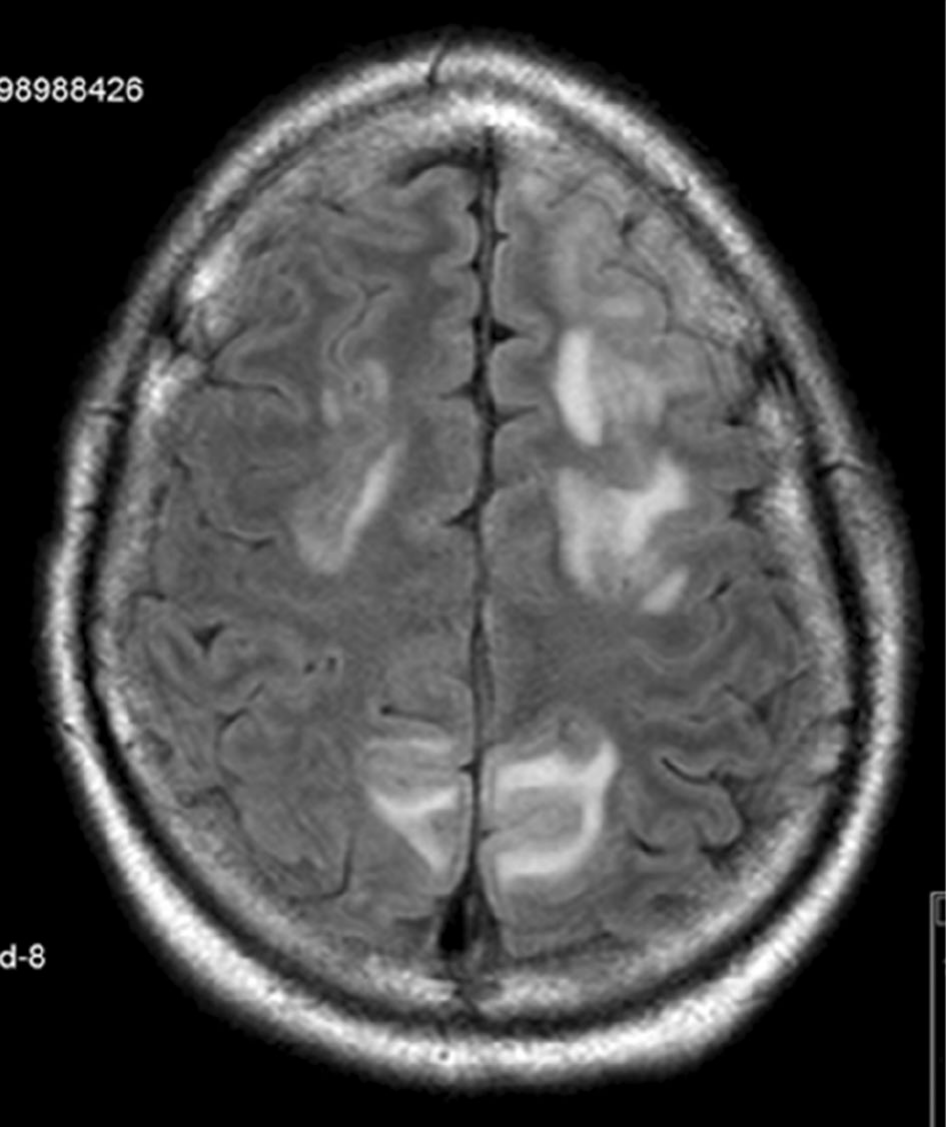
Posterior reversible encephalopathy syndrome in a young patient with T-cell lymphoblastic lymphoma, presenting with headache and seizure after first chemotherapy cycle (pegylated l-asparaginase, vincristine, doxorubicin, and methotrexate): bilateral asymmetrical parieto-frontal hyperintensities on FLAIR sequence

PRES should be considered in patients with hematological malignancies treated with chemotherapy or immunotherapies, presenting with headache, visual disturbances, seizures, focal signs, or encephalopathy. Usually, brain CT is normal, while MRI T2/FLAIR sequences, but not DWI, show hyperintense symmetric lesions, typically located on the occipito-parietal regions, but which can also have a water-shed appearance, or less classic locations in the superior frontal lobe and even in the basal ganglia or brain stem (Fig. 5).

These lesions are due to vasogenic edema and are usually reversible in 2–4 weeks. In severe cases, cytotoxic edema may develop, causing irreversible lesions. Brain hemorrhages may also appear. Besides chemo/immunotherapy, hypertension, sepsis, and uremia are additional causative factors for PRES. The prognosis is in general good, with complete recovery, but a few patients may die or be left with neurological deficits or epilepsy. Treatment includes correcting any ionic or metabolic imbalance and infection, controlling hypertension, and transient or permanent withdrawal of the causative medications [81]. The chemotherapy scheme may be revised, but can be resumed, as PRES rarely recurs on re-exposure to the initial causative agent.

### Methotrexate-induced stroke-like episodes

Methotrexate (MTX) is a folic acid antagonist which is used in the treatment of acute leukemia to prevent meningeal and brain leukemic infiltration. A rare subacute complication of MTX manifests clinically as stroke, with sudden onset of aphasia and/or hemiparesis and/or a disturbance of consciousness, 1–13 days after MTX treatment [82–84]. Fibrin degradation products and D-Dimers are normal. MR shows multiple, bilateral lesions on DWI/ADC. There is usually a rapid improvement in a few days, with clearing of the DWI lesions, and in general, no permanent lesions are visible on FLAIR sequences. The syndrome may uncommonly recur on subsequent MTX administrations. The pathophysiology of these episodes is not well known, but probably involves multiple mechanisms, in particular hyperhomocysteinemia. Dextromethorphan was used in one patient [82] and adaravone and mannitol in four others [83], but no conclusions can be made regarding its efficacy, due to the lack of controls and the spontaneous recovery seen in most patients.

### Chimeric antigen receptor (CAR) T-cell therapy

Chimeric antigen receptor (CAR) T-cell therapy is a novel and effective cellular immunotherapy using genetically engineered, tumor-specific autologous T cells, currently used for relapsed/refractory B-cell acute lymphoblastic leukemia, and aggressive B-cell non-Hodgkin’s lymphoma. One of the more frequent side-effects of this CAR T-cell therapy is a severe neurotoxic syndrome (immune effector cell-associated neurotoxicity syndrome—ICANS). Patients with neurologic symptoms following CAR T-cell infusion suspected to have ICANS can suffer from other entities such as ischemic stroke, PRES, and CVT. It is crucial to consider causes other than ICANS in patients who experience neurologic symptoms after CAR T-cell treatment [85, 86].

### Hematopoietic stem cell transplantation

PRES (4.29%) and stroke (3.78%) are among the commonest neurological complications after allogenic hematopoietic stem cell transplantation for the curative treatment of malignant hematological disorders. The risk of stroke is 15 × higher than in the general population [87]. Three recent series [88–90] found low frequencies of both of PRES (4%) and stroke (< 1% in the first 100 days [79] to 10% in longer follow-up). While PRES usually occurs early (up to 100 days) after transplantation, ischemic stroke may occur early, late (up to 2 years), or very late, while intracerebral hemorrhage happens predominantly early on [88]. In one of the series, all but 1/15 strokes were hemorrhagic [89]. Ten of these patients died, despite surgical evacuation in five of them. Age, acute graft-vs-host disease grade II/III–IV, extensive graft-vs-host disease, transfusion-dependent thrombocytopenia, and delayed platelet engraftment were risk factors for these cerebrovascular complications. Stroke is associated with reduced survival. Mortality is high when the intracerebral hemorrhage is associated with severe thrombocytopenia [87]. Most of the cases of PRES are due to the administration of a calcineurin Inhibitor (cyclosporine, tacrolimus). PRES has a lower occurrence in the auto-transplant population (0.86%) [87].

## Conclusions and future directions

Hematological diseases are a rare cause of stroke, but cerebrovascular complications are frequent in patients with hematological diseases. Cerebrovascular disease has a negative impact on their prognosis. The complex management of stroke in the context of the different hematological diseases requires close cooperation between hematologists, neurologists, neuroradiologists, and sometimes neurosurgeons. Acute stroke treatment is often a dual emergency, as the patient needs both immediate treatment for the neurovascular disturbance and for the underlying hematological condition. The evidence supporting decisions in stroke acute treatment and secondary prevention varies in quality from high (e.g., randomized-controlled trials of transfusions and of hydroxyurea in sickle-cell disease, where recurrent stroke was the primary outcome) to very low (e.g., thrombectomy in acute stroke in sickle-cell disease or in CVT associated with ALL). Due to the low frequency of some diseases (e.g., POEMS, PNH, and TTP) or association of conditions (e.g., stroke and multiple myeloma), it is unlikely that randomized trials will be performed to answer specifically questions on the optimal acute stroke treatment or secondary stroke prevention. New evidence will probably come from subgroup/secondary analysis of trials looking at treatment or prevention of all thrombotic complications of the individual hematological diseases. Descriptive analysis of “big” administrative data will also be very useful to inform on outcome of prespecified interventions for stroke in uncommon scenarios. New chemo and immune therapies for hematological diseases in the future will have to be monitored for potential associations with stroke, as well as other neurological complications such as PRES and ICANS.

## Data Availability

Available upon reasonable request.

## Code availability

Available upon reasonable request.
